# Two center experience of capsule endoscopy in Iran: Report on 101 cases

**DOI:** 10.12688/f1000research.11288.2

**Published:** 2018-02-12

**Authors:** Fariborz Mansour-Ghanaei, Morteza Asasi, Farahnaz Joukar, Rahmatollah Rafiei, Alireza Mansour-Ghanaei, Ehsan Hajipour-Jafroudi

**Affiliations:** 1 Gastrointestinal and Liver Diseases Research Center, Razi Hospital, Guilan University of Medical Sciences, Rasht, Iran; 2 Caspian Digestive Diseases Research Center, Guilan University of Medical Sciences, Rasht, Iran; 3 Department of Internal Medicine, School of Medicine, Islamic Azad University, Najafabad, Isfahan, Iran; 4 Shahid Beheshti University of Medical Sciences, Tehran, Iran; 5 Tehran University of Medical Sciences, Tehran, Iran

**Keywords:** Capsule Endoscopy, Diagnostic Imaging, Crohn disease, Gastrointestinal Diseases, Double-Balloon Enteroscopy

## Abstract

**Background:** Capsule endoscopy (CE) is a minimally invasive method for the visual examination of the small intestine, which may be for the evaluation and follow-up of patients with Crohn's disease. It can also be used to look at mucosal inflammation.

**Methods:** This cross sectional study was used to determine the diagnostic efficacy of the CE system by performing a cross-sectional study of cases from 2011-2014. This study involved a total of 101 Iranian patients who were referred to the gastrointestinal and liver diseases outpatient clinics in Guilan (northern Iran) and in Isfahan (central Iran) for complaints of gastrointestinal problems. For all patients, definitive diagnosis had failed with the use of other diagnostic tools and CE was performed. Descriptive analysis was used. The patient population was represented by men and women equally, and the mean age of the patients was 42.3 ± 17.2 years (range: 16-89 years).

**Results:** The final diagnoses were: non-specific enteritis (30.6%), Crohn's disease (20.7%), ulcers caused by aspirin or non-steroidal anti-inflammatory drugs (8.9%), mucosal erosion (5.9%) and angioectasia (4.9%); nearly 10% of the patients had normal findings. Analysis of the distribution of chief presenting complaints with patients stratified by the final diagnosis of Crohn's disease showed that the most frequently presented chief complaint was abdominal pain 42.9% and the least frequently presented chief complaint was diarrhea (4.8%).

**Conclusions:** Small bowel evaluation by CE was well tolerated and capable of diagnosing Crohn's disease and gastrointestinal bleeding in patients who failed other diagnostic tests.

## Introduction

Capsule endoscopy (CE) is a relatively new imaging technique used to visualize, evaluate and diagnose the gastrointestinal tract in a non-invasive manner. While it has proven to be feasible and well tolerated for examination of the small bowel, CE has not yet emerged as an efficacious alternative to traditional endoscopy for the esophagus, stomach, duodenum or colon ^1^. Yet, its applications in small bowel continue to evolve and advance.

The recently developed double-balloon enteroscopy system is based upon the classical endoscopic approach of inserting a flexible endoscope, which is covered by a special tube that has two balloons at the end ^1, 2^. Inflation and deflation of the balloons allow for further advancement into the bowel and more extensive viewing of the mucosa. Although this device is able to facilitate biopsy taking and remedial actions, its application is very time consuming and it may not be feasible to examine the small bowel completely. CE is another recently developed technique and boasts the distinct advantage of being capable of providing endoscopic evaluation of the small bowel completely ^3, 4^.

Compared to traditional endoscopy, CE has a higher sensitivity because it allows for examination of otherwise inaccessible areas of the small bowel and facilitates the operator’s ability to detect changes and diagnose disease ^5, 6^. Moreover, the latest advancement in the use of video capsule allows operators to visualize the complete small bowel ^7^. During its 4-h to 6-h trek through the small bowel, a capsule will transfer captured images wirelessly to an external receiver that is worn by the patient. These images are of high quality and comparable to those taken by ordinary scopes ^8, 9^.

CE has particularly high sensitivity and specificity for diagnosis of gastrointestinal lesions. As such, the most popular application of CE has emerged as determining the causes of gastrointestinal symptoms, such as gastrointestinal bleeding, malabsorption and unspecified abdominal pain; the main diagnoses are small bowel tumors, angiodysplasia and inflammatory diseases, such as Crohn’s disease, infectious enteritis, celiac sprue and ulcers caused by use of non-steroidal anti-inflammatory drugs ^1, 2, 6^. Several studies have also shown the utility of CE for diagnosis of celiac disease and its complications ^10– 12^, and for detection of polyps to screen for Peutz-Jeghers syndrome and familial adenomatous polyposis ^2, 5, 7^. Hence, video CE would represent an alternative option for patients who are unable or unwilling to undergo esophagogastroduodenoscopy ^13^. Contraindications to CE include known or suspected bowel obstruction, strictures or fistulas (which have been detected by other clinical imaging or tests prior), cardiac pacemakers, implanted electro-myocardial tools and swallowing disorders ^14^.

The OMOM CE System, manufactured by Jinshan Science & Technology (Group) Co., Ltd (Chongqing, China), provides good quality images and is available at a reasonable price, making it a feasible option for smaller healthcare institutes and/or countries with developing economies ^15^. In this study, we performed OMOM CE to diagnose gastrointestinal diseases in adult patients referred to two gastroenterology clinics in the Guilan and Isfahan provinces of Iran for evaluation of various gastrointestinal complaints between January 2011 and February 2014. The study was performed in order to distinguish the proficiency of OMOM CE and highlight its importance to other gastroenterologists.

## Methods

This cross-sectional study included patients that were referred to the gastrointestinal and liver disease outpatient clinic of Razi Hospital of the Guilan University of Medical Sciences (GUMS) in Rasht, north of Iran, and Dr Rafiei’s gastroenterology clinic in Isfahan (private clinic), with a nonspecific age criteria and symptoms such as gastrointestinal bleeding of unknown origin; abdominal pain; chronic diarrhea; suspected inflammatory bowel disease; iron deficiency anemia; suspected tumors and/or polyps; malabsorption; unintentional weight loss without any diagnosis by other type of conventional evaluation. Patients were denied enrollment if any of the following were present: known or suspected bowel obstruction; strictures or fistulas detected by prior clinical imaging or tests; cardiac pacemakers; implanted electro-myocardial tools; swallowing disorders. Also, small bowel series were conducted for all patients.

All the examinations were carried out under the following conditions. Iron supplementation was stopped 3 d before the examination. Consumption of antacids or bismuth components, which are known to coat the camera lens, was discontinued 1d before the examination. Starting at 8 am on the day before the test, the patient was permitted only clear liquids with a light breakfast. The patients consumed one dose (70 g) of polyethylene glycol laxative mixed in 250 mL water at 4 pm on the day before the test. All the patients were fully fasting starting at 8 pm on the day before the test, with the exception of any critical medications, which were given with sips of water. The procedure was performed at 8 am on the test day.

The following data was collected for each study participant: age; sex; clinical manifestation; chief complaint; preliminary tests performed, including hemoglobin and stool (ova & parasite, occult blood); final diagnosis with CE.

### Data analysis

The acquired images were reviewed by the two coordinate gastroenterologists. For the descriptive analyses, quantitative variables are expressed as means with standard deviation values; data of analyses of qualitative variables are expressed as frequency and percentages.

### Ethical statement

The Medical Ethics Committee of GUMS (P/3/132) has approved the study design, protocol and informed consent procedure. All measurements were performed based on ethical guidelines of the 1975 Declaration of Helsinki. Informed written consent was obtained from each patient. All patients consented to the study.

## Results

A total of 101 patients were enrolled in the study, based upon the inclusion and exclusion criteria. The study population was represented equally by the two sexes (48.5% male), and the mean age of the patients was 42.3 ± 17.2 years (range: 16–89 years). The most frequent chief complaints that led to evaluation by CE were abdominal pain and anemia, accounting for 40.6% and 21.8% of the cases, respectively ( Table 1 and Table 2). In patients with Crohn’s disease, the most frequent chief complaint was abdominal pain 42.9% and the least frequent was diarrhea (4.8%).

**Table 1.** Chief complaints of patients diagnosed by OMOM capsule endoscopy.

Chief compliant	*n* (%)
Abdominal pain	41 (40.6)
Diarrhea	9 (8.9)
Anemia	22 (21.8)
Occult blood in stool	9 (8.9)
Abdominal pain + diarrhea	12 (11.9)
Abdominal pain + anemia	8 (7.9)

**Table 2.** Findings of OMEM capsule endoscopy for the 101 patients in this study.

Final diagnosis	*n* (%)
Crohn’s disease	21 (20.7)
Angioectasia	5 (4.9)
Non-specific enteritis	31 (30.6)
Ulcers caused by aspirin or NSAIDs	9 (8.9)
Small intestine tumors	2 (1.9)
Gastropathy	12 (11.8)
Celiac disease	1 (1.0)
Tapeworm	1 (1.0)
Small intestine polyps	2 (1.9)
Small intestine mucosal erosion	6 (5.9)
Gastric polyp	1 (1.0)
No disease	10 (9.9)

NSAIDs: non-steroidal anti-inflammatory drugs.

**Figure 1.**
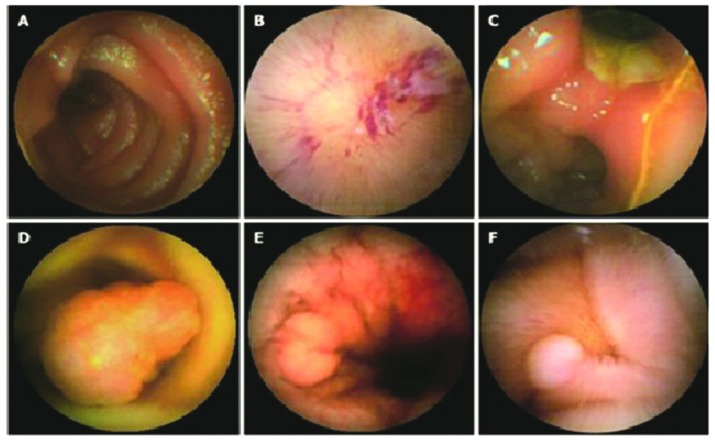
Small bowel images captured by OMOM capsule endoscopy. ( **A**) Normal small bowel mucosa; ( **B**) Angioectasia; ( **C**) Ulcer; ( **D**) Tumor; ( **E**) Crohn’s disease; ( **F**) Polyp.

When the patients were grouped by sex, Crohn’s disease was the most frequent diagnosis in both males (61.9%) and females (38.1%). When the patients were grouped by age, Crohn’s disease was the most frequent diagnosis for both young adults (<30 years: 61.9%) and middle-aged adults (30–50 years: 28.6%), and angioectasia was the most frequent in patients > 50 years in age. Some of the observed results are shown in Figure 1.

Data for all the variables collected for each study participant in SPSS and Excel format, i.e. age, sex, gender, clinical manifestation, preliminary tests performed (DX-primary), final diagnose with CE (DX-End) Click here for additional data file. Copyright: © 2018 Mansour-Ghanaei F et al. 2018 Data associated with the article are available under the terms of the Creative Commons Zero "No rights reserved" data waiver (CC0 1.0 Public domain dedication).

## Discussion

CE was first described by its inventor Gavriel Iddan in 1981, and since has become a commonly applied clinical tool for evaluation of gastrointestinal disorders and diagnosis of gastrointestinal diseases. The current study, however, represents the first of its kind to be carried out in hospitals and patients in Iran. A similar study was conducted previously in New York in the United States ^16^, but the patient population was slightly skewed towards the female sex (57% *vs* 48% in our study) and the mean age of patients was higher (61 years *vs* 43 years in our study). Moreover, the previous study focused on patients who had been referred for gastrointestinal bleeding, whereas our patients were referred for a variety of common gastrointestinal complaints.

The OMOM CE system is used throughout China and in many European and other Asian countries. The OMOM capsule is slightly larger than another popular small bowel capsules, such as the PillCam capsule, but our patients experienced no problems with its use. Similar to the PillCam capsule’s battery, the OMOM capsule’s battery can last 8 h. Two exclusive features of CE, as compared to traditional endoscopy, are its ability for real-time imaging and recording of information ^8^. A new type of capsule, with a smaller size and a new shape, has just recently been developed and released to market by the OMOM manufacturer in China, and this will likely advance the use of CE even further.

The current study investigated patients treated with the older OMOM capsule, exclusively. The most frequent chief complaints that led to CE evaluation were abdominal pain, either in isolation or accompanied by diarrhea; this complaint distribution fits with the frequent diagnosis of Crohn’s disease (28.6 % of patients that had abdominal pain and diarrhea, and 42.9% that had abdominal pain in isolation). These findings were different from those in the study by Ruuska *et al.* ^17^, where abdominal pain in isolation was the most frequent chief complaint (42%), followed by diarrhea (17%) and weight loss (5%). Similarly, a study Mohan *et al.* ^18^ examined the role of CE in evaluation of patients with suspected small bowel bleeding and identified the most frequent chief complaint as abdominal pain (43%) and a small portion of patients with the chief complaint of weight loss (6%).

CE is commonly used to evaluate cases of occult gastrointestinal bleeding, such as that which underlies iron deficiency anemia, Crohn’s disease and small intestinal tumors; its utility has also been proven for evaluating polyposis syndromes and refractory malabsorptive syndromes, such as celiac disease ^6^. According to the literature, the most common uses of OMOM CE in China involve patients with occult gastrointestinal bleeding and who present with complaints of abdominal pain and chronic diarrhea ^6^. CE findings of inflammation in the small bowel are indicative of inflammatory bowel disease, and help clinicians to diagnose Crohn’s and other inflammation-related disorders ^19^. A 2003 study of the technical performance and efficiency of CE reported by Mylonaki *et al.* ^20^, which investigated 50 patients with gastrointestinal bleeding undiagnosed by colonoscopy and gastroscopy, found the diagnosis rate of occult gastrointestinal bleeding was ~43%.

Incidence of Crohn's disease is increasing among the Chinese ^17^ and Iran is facing a similar situation ^21^. Zheng and colleagues ^22^ warned that, while Crohn’s disease incidence and prevalence rates in China are still lower than the rates reported from Western countries and even Asian industrialized countries, they are increasing rapidly. Previous studies, including one in 2007 by Fidder *et al.* ^23^ have recommended the use of CE to diagnose Crohn’s disease, based upon the reported evidence of its ability to detect the disease condition in a very small percentage of patients (0 to 4%).

In our study, two patients were diagnosed with small intestinal tumors. The 2010 study by Jung Wan Han *et al.* ^7^ indicated that CE was capable of detecting particularly challenging or otherwise undetectable tumors in the small bowel. Without CE, many of these tumors may only become detectable by the other available technologies at the later or last stages of cancer, when therapies are less efficacious or feasible. Those authors also reported that CE has sufficiently higher diagnostic yield and sensitivity for definitive small bowel tumors.

In the present study, one patient was diagnosed with a tapeworm by CE. Several other studies have reported hookworm detection by CE, including a report of 26 Chinese patients with occult gastrointestinal bleeding published by Liao *et al.* ^17^, in which 3.4% of the patients were diagnosed with hookworm. Another 6 case reports, 4 of which were from Asian countries, showed hookworm as the etiology of gastrointestinal bleeding, as diagnosed by CE ^19, 24– 28^. Although capsule retention is one of the reported side effects of capsule endoscopy in patients with Crohn’s disease ^29^, in our study this issue was not observed.

## Conclusions

Medical science is continually looking for ways to eliminate any aggressive (invasive and/or risky) methods of evaluating the body, and the CE method is an excellent way to detect small bowel diseases when traditional endoscopy cannot detect the problems. The OMOM CE system in particular, is a valuable device for small bowel evaluation because of the small size of its capsule, high-resolution images and low price, which supports its use in healthcare settings across the globe and in the diagnosis of common gastrointestinal complaints, especially Crohn’s disease.

## Data availability

The data referenced by this article are under copyright with the following copyright statement: Copyright: © 2018 Mansour-Ghanaei F et al.

Data associated with the article are available under the terms of the Creative Commons Zero "No rights reserved" data waiver (CC0 1.0 Public domain dedication). 

Dataset 1: Data for all the variables collected for each study participant in SPSS and Excel format, i.e. age, sex, gender, clinical manifestation, preliminary tests performed (DX-primary), final diagnose with CE (DX-End). doi, 10.5256/f1000research.11288.d178638 ^30^
