# Supplementary material for: Two center experience of capsule endoscopy in Iran: Report on 101 cases
Source: F1000Res. 2018 Feb 12;6:1765. Originally published 2017 Sep 27. [Version 2] doi: 10.12688/f1000research.11288.2 (PMC5814745; doi:10.12688/f1000research.11288.2)
Supplement: Peer review history

# Peer review: Referee response for version 2

**DOI:** 10.5256/f1000research.14976.r30758

**Published:** 2018-02-14

**Authors:** Marco Pennazio

**Affiliations:** 1 Gastroenterology and Hepatology Department of Medicine, San Giovanni Battista University Teaching Hospital, Turin, 10123, Italy

The paper is now acceptable and can be indexed.

I have read this submission. I believe that I have an appropriate level of expertise to confirm that it is of an acceptable scientific standard.

# Peer review: Referee response for version 1

**DOI:** 10.5256/f1000research.12178.r29793

**Published:** 2018-01-16

**Authors:** Eva Niv

**Affiliations:** 1 Capsule Endoscopy Unit, Department of Gastroenterology, Tel-Aviv Sourasky Medical Center, Tel Aviv, Israel

The paper is well-written and presents interesting data regarding the results of capsule endoscopy in Iran. It presents data about the prevalence of different small bowel pathologies in Iran population. I support the publication of this paper. For my opinion, there is no need in changes, besides minor language corrections.

I have read this submission. I believe that I have an appropriate level of expertise to confirm that it is of an acceptable scientific standard.

# Peer review: Referee response for version 1

**DOI:** 10.5256/f1000research.12178.r28927

**Published:** 2017-12-12

**Authors:** Marco Pennazio

**Affiliations:** 1 Gastroenterology and Hepatology Department of Medicine, San Giovanni Battista University Teaching Hospital, Turin, 10123, Italy

This is a descriptive report on the use of capsule endoscopy for the investigation of a quite heterogenoeous population.

 I suggest to better specify:

1. In patients with Crohn's disease: was this diagnosis suspected before CE or Crohn's disease was already Known before CE
2. specify if,  in patients with known Crohn's disease before CE, the subsequent endoscopic examination turned out to be negative
3. if the diagnosis of Crohn's disease was only suspected before CE, please specify how the diagnosis was made on the basis of the capsule findings
4. did the authors observed any complication after CE (ie capsule retention)?
5. in the discussion please underline that patients with known Crohn's disease are at increased risk of capsule retention and therefore every effort (patency capsule; MRE before CE) should be made in order to minimize this risk.
6. with reference to this last point please quote: Pennazio M, Spada C, Eliakim  Small-bowel capsule endoscopy and device-assisted enteroscopy for diagnosis and treatment of small-bowel disorders: European Society of Gastrointestinal Endoscopy (ESGE) Clinical Guideline. Endoscopy 2015;47(4):352-76.

I have read this submission. I believe that I have an appropriate level of expertise to confirm that it is of an acceptable scientific standard, however I have significant reservations, as outlined above.

# Response

**Published:** 2018-01-15

**Authors:** Farahnaz Joukar

**Affiliations:** Guilan University of Medical Sciences, Iran

Thank you very much for the insightful review comments. Here are the related point-by-point replies according to the comments. 

1. The diagnosis of questionable patients with Crohn’s disease before capsule endoscopy was based on existence of abdominal pain, anemia and the physician clinical suspicious
2. The capsule endoscopy findings of Crohn’s disease among patients were after exclusion of NSAID consumption and diagnosis of parasitic infections. Furthermore, the patient’s treatment response was considered as the diagnosis confirmation.
3. After capsule endoscopy no side effects were reported.
4. It is added in the text as “Although capsule retention is one of the reported side effects of capsule endoscopy in patients with Crohn’s disease ^30^, in our study this issue was not observed”.
5. In the last part of the methods section we have added “Also, small bowel series were conducted for all patients”.
